# Is obstructive sleep apnea a driver of cancer and chronic disease risk? A real-world analysis of over 3 million patients

**DOI:** 10.1186/s12890-026-04329-5

**Published:** 2026-05-12

**Authors:** Simon Bigus, Nils Lucca Kern, Tim Lukas Elter, Max Heiland, Robert Preissner, Saskia Preissner

**Affiliations:** Department of Oral and Maxillofacial Surgery, Charité – Universitätsmedizin Berlin, Corporate Corporate Member of Freie Universität Berlin and Humboldt-Universität zu Berlin, Augustenburger Platz 1, Berlin, 13353 Germany

**Keywords:** Obstructive Sleep Apnea, Cancer risk, TriNetX, Cardiometabolic disease, Chronic obstructive pulmonary disease, Asthma, Diabetes mellitus, Real-world data

## Abstract

**Background:**

Obstructive sleep apnea (OSA) is a common sleep-related breathing disorder linked to substantial cardiovascular, pulmonary and metabolic morbidity. Recent observations have raised the question that OSA may also contribute to cancer development. This study aimed to evaluate the association between OSA and the 5-year incidence of cancer, cardiometabolic, pulmonary and metabolic disorders in a large real-world hospitalized population.

**Methods:**

A retrospective analysis of the TriNetX research database was conducted. Two cohorts of 1,524,190 adults with and without OSA, matched for age and sex, were analyzed for incident cancer, chronic ischemic heart disease, chronic obstructive pulmonary disease (COPD), asthma, overweight/obesity, and diabetes mellitus. Risk estimates, risk differences, risk ratios (RR), and odds ratios were calculated; cancer risk was additionally analyzed using Kaplan–Meier curves and log-rank tests. Significance was indicated by *p* < 0.05.

**Results:**

OSA was associated with significantly higher 5-year incidences of all predefined outcomes, including cancer (RR 1.40), chronic ischemic heart disease (RR 1.36), COPD (RR 1.64), asthma (RR 1.96), diabetes mellitus (RR 1.51), and overweight/obesity (RR 2.70) (*p* < 0.001).

**Conclusions:**

In this large real-world analysis, OSA was associated with increased 5-year risks of incident cancer and major cardiovascular, pulmonary, and metabolic diseases. The magnitude of cancer risk was comparable to that of non-malignant chronic conditions, supporting the concept of OSA as a systemic disorder characterized by multisystem morbidity.

## Background

Obstructive sleep apnea (OSA) is the most common sleep-related breathing disorder, with a global prevalence of approximately 54% [1]. Risk factors for OSA include higher body mass index (BMI), older age, male sex, alcohol consumption and smoking [1, 2]. OSA is characterized by recurrent episodes of partial or complete upper airway obstruction during sleep, resulting in intermittent apnea and hypopnea, sleep fragmentation, and nocturnal hypoxemia [3, 4]. Common clinical manifestations include snoring or choking during sleep, excessive daytime sleepiness, fatigue, morning headaches, and cognitive impairment.

OSA is increasingly recognized as a systemic disorder with a wide range of adverse health consequences. Numerous studies have demonstrated associations between OSA and cardiovascular disease, metabolic dysfunction, pulmonary disease, depression, adverse perioperative outcomes and Parkinson disease [3–12]. From a cardiovascular perspective, OSA has been robustly linked to systemic hypertension, coronary artery disease, heart failure, atrial fibrillation, and stroke [13–15]. Similarly, OSA is closely associated with metabolic disease, including insulin resistance, type 2 diabetes overweight and obesity, through mechanisms involving intermittent hypoxemia, sleep fragmentation, inflammation, hormonal dysregulation and activation of neurohumoral stress pathways [16, 17]. OSA is also associated with a range of pulmonary disorders including chronic obstructive pulmonary disease (COPD), pulmonary hypertension or asthma, which further contribute to its systemic disease burden [18–25]. In recent years, attention has also turned to a potential link between OSA and an increased risk of cancer [26]. A meta-analysis demonstrated that severe nocturnal hypoxemia, a hallmark of OSA, significantly increased the risk of cancer incidence and nearly tripled the risk of all-cause cancer mortality [27].

Despite the well-established association between OSA and a wide range of chronic diseases, an important gap remains in the existing literature: long-term risk estimates of these outcomes are poorly defined. Most available data report relative measures of association without offering standardized, time-specific estimates [28–30]. As a result, robust and comparable 5-year risk estimates for clinically relevant outcomes, including asthma, COPD, chronic ischemic heart disease, diabetes, overweight, obesity, and cancer, remain largely unavailable [31, 32].

To address this gap, we conducted a large-scale epidemiological analysis using real-world data from the TriNetX network, a global federated database of electronic health records. The objective of this study was to quantify and compare 5-year risks of incident cancer and cardiometabolic, pulmonary and metabolic diseases in hospitalized adults with and without OSA, using a standardized, propensity score–matched approach in a broad real-world approach.

## Methods

A retrospective cohort study was conducted using data from the TriNetX Research Network, a global federated health platform that aggregates de-identified electronic medical records from over 100 healthcare organizations (HCOs) in compliance with the Health Insurance Portability and Accountability Act (HIPAA). All data were de-identified in accordance with the HIPAA Privacy Rule (§ 164.514) through formal expert determination. As no identifiable patient information was accessed, this study did not require institutional review board approval or informed consent. The study design, conduct, and reporting adhered to the STROBE statement.

The TriNetX network was queried on October 28, 2024. Two cohorts of hospitalized patients were identified based on inpatient encounters and the presence or absence: patients with a documented OSA diagnosis (ICD-10: G47.33) and a control cohort without OSA. Both cohorts were restricted to at least one inpatient encounter. Propensity score matching was performed to balance cohorts for age and male sex. Additional clinical and lifestyle-related variables were not included in the matching procedure. Therefore, potentially relevant clinical and lifestyle-related confounders, such as obesity, smoking, and other comorbidities, were not accounted for in the matching procedure.

The index event was defined according to the cohort-defining criteria. For the OSA cohort, the index event was the first inpatient encounter with a recorded OSA diagnosis (ICD-10: G47.33) and a corresponding inpatient visit code. No information on OSA severity, such as apnea–hypopnea index, oxygen desaturation burden, or treatment status, was available within the applied study design. For the non-OSA cohort, the index event was the first inpatient encounter meeting the visit criterion. TriNetX restricts index events to those occurring within the previous 20 years; therefore, patients with index events ≥ 20 years before the analysis date are excluded.

**Fig. 1:**
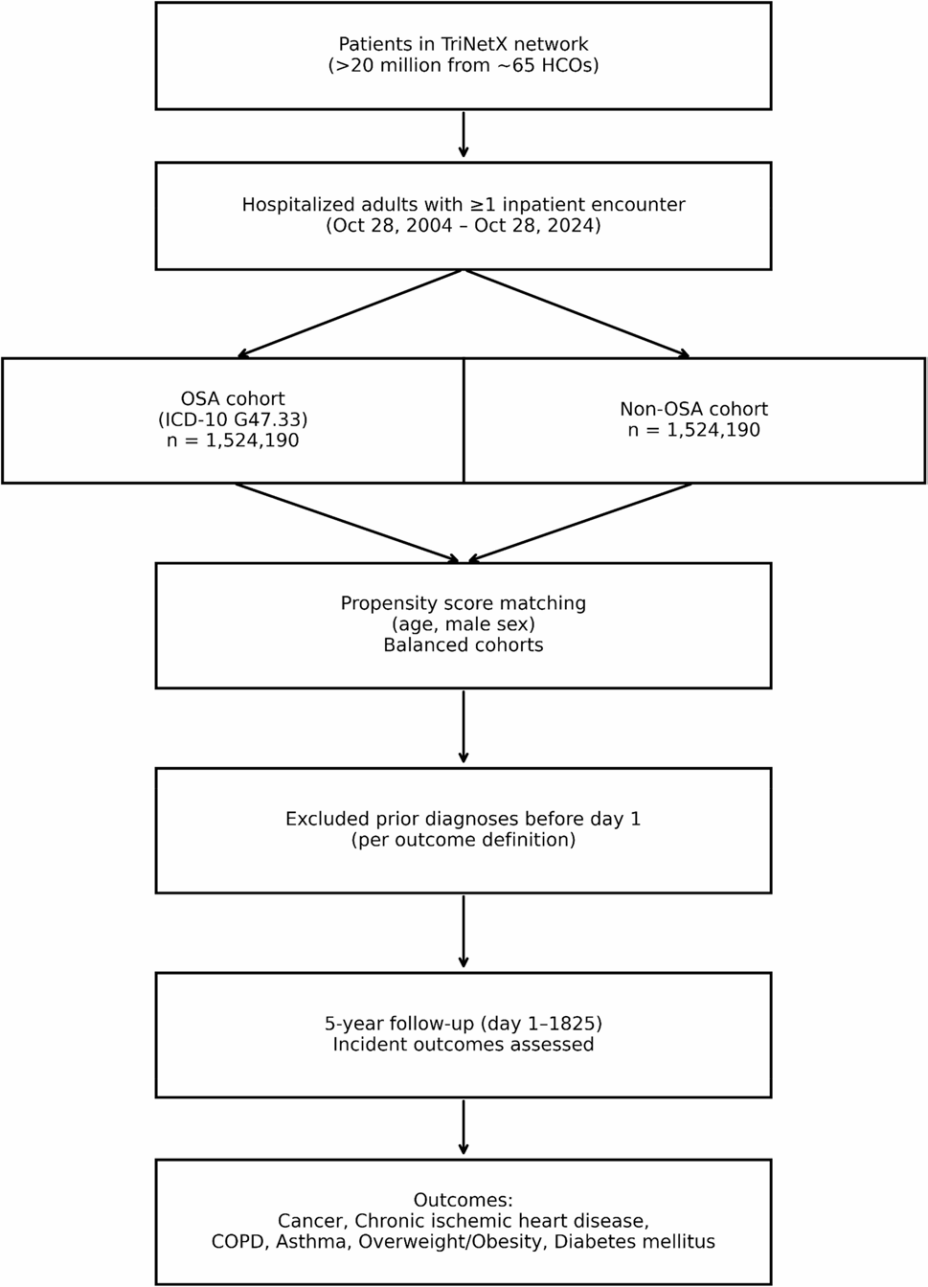
STROBE Flow Diagram of cohort selection and outcome assessment

The study workflow is illustrated in Fig. 1.

### Outcomes

Six clinically relevant incident outcomes were assessed within a 5-year observation window following the index event, based on ICD-10-CM codes: cancer (neoplasms, C00–D49), chronic ischemic heart disease (I25), chronic obstructive pulmonary disease (J44), asthma (J45), overweight and obesity (E65–E68), and diabetes mellitus (E08–E13). For each outcome, patients with any documented diagnosis of the respective condition prior to day 1 of the observation window were excluded, ensuring the assessment of incident rather than prevalent disease. An outcome was considered present if the first qualifying diagnosis occurred between day 1 and day 1,825 (5 years) following the index event. Patients were included if their index event occurred between October 28, 2004, to October 28, 2024. All definitions followed the standardized TriNetX clinical ontology to ensure consistency across data-contributing healthcare organizations.

### Statistical analysis

All statistical analyses were conducted within the TriNetX platform. For each outcome, the cumulative 5-year risk was calculated as the proportion of patients with at least one incident event within the observation window. Comparative measures included risk difference, risk ratio, and odds ratio, each reported with 95% confidence intervals (95% CI) based on platform-generated binomial estimates. For cancer, additional time-to-event analyses were performed using Kaplan–Meier curves, log-rank tests, and Cox proportional hazards models. The proportional hazards assumption was assessed within the platform, and hazard ratio estimates were interpreted cautiously where this assumption appeared to be violated. For the remaining outcomes, the analysis focused on cumulative 5-year risk estimates, including risk differences, risk ratios, and odds ratios, to provide a standardized comparison across the different evaluated outcomes. Hazard ratios with 95% CI were derived from proportional hazards models implemented within TriNetX. All analyses were performed on the propensity-score–matched cohorts, ensuring balanced baseline characteristics. A default two-sided significance threshold of *p* < 0.05 was applied throughout.

## Results

About 65 HCOs responded with over twenty million patients for the two cohorts. Propensity score matching resulted in two homogeneous cohorts of 1,524,190 patients, each with excellent covariate balance. Age at index was identical in both groups (56.3 ± 19.4 years in each cohort), and sex distributions were perfectly matched (52.4% male in both groups). Standardized differences for these key variables were effectively zero, indicating successful elimination of measurable baseline imbalances.

### Risk analysis

**Fig. 2:**
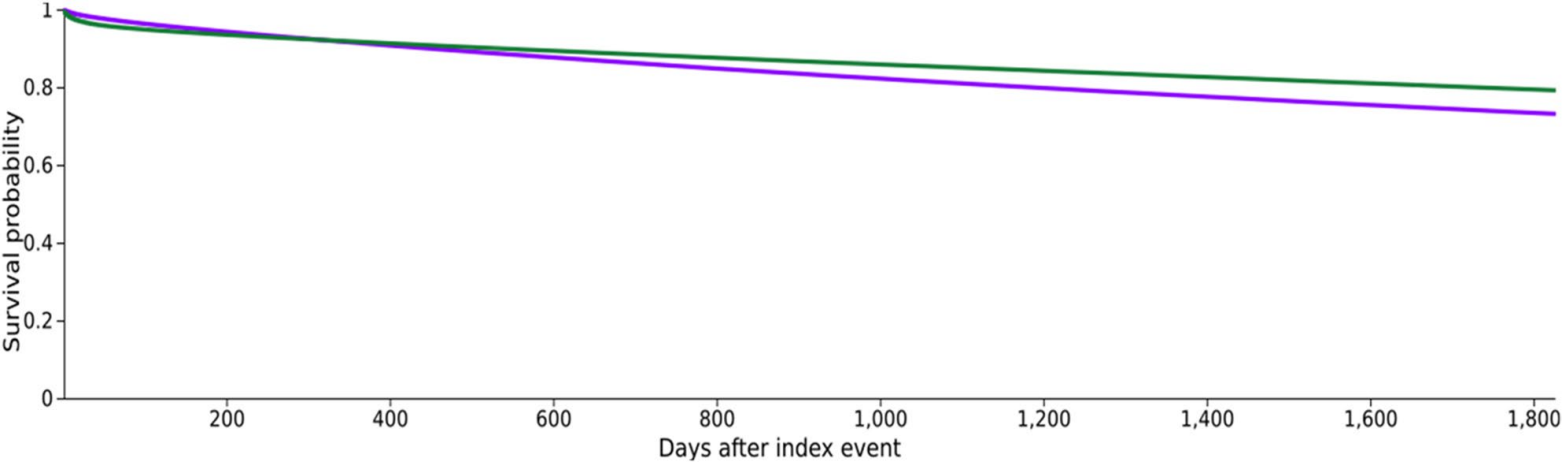
Kaplan–Meier Curves for 5-Year Cancer Incidence in Patients With and Without Obstructive Sleep Apnea. Kaplan–Meier Curves for Incident Cancer in OSA (purple) vs. non-OSA patients (green)

For cancer, 970,170 patients with OSA and 1,220,689 control patients were included. Due to the outcome prior to the time window, 554,020 patients with OSA and 303,501 patients in the control cohort were excluded. Incident cancer occurred in 161,075 patients with OSA (risk 0.166) and 145,311 control patients (risk 0.119), resulting in a risk difference (RD) of 0.047 (95% CI: 0.046–0.048; z = 99.606; *p* < 0.001), a risk ratio (RR) of 1.395 (95% CI: 1.386–1.404), and an odds ratio (OR) of 1.473 (95% CI: 1.462–1.485). Kaplan–Meier analysis indicated a significantly lower probability of remaining cancer-free in the OSA cohort (73.14% vs. 79.20%; log-rank χ² = 3366.244; *p* < 0.001), with a hazard ratio of 1.233 (95% CI: 1.224–1.242). However, the test of the proportional hazards assumption was statistically significant (χ² = 2277.566, df = 1, *p* < 0.001), indicating that this assumption may have been violated (Fig. 2). Therefore, the hazard ratio should be interpreted with caution.

**Fig. 3:**
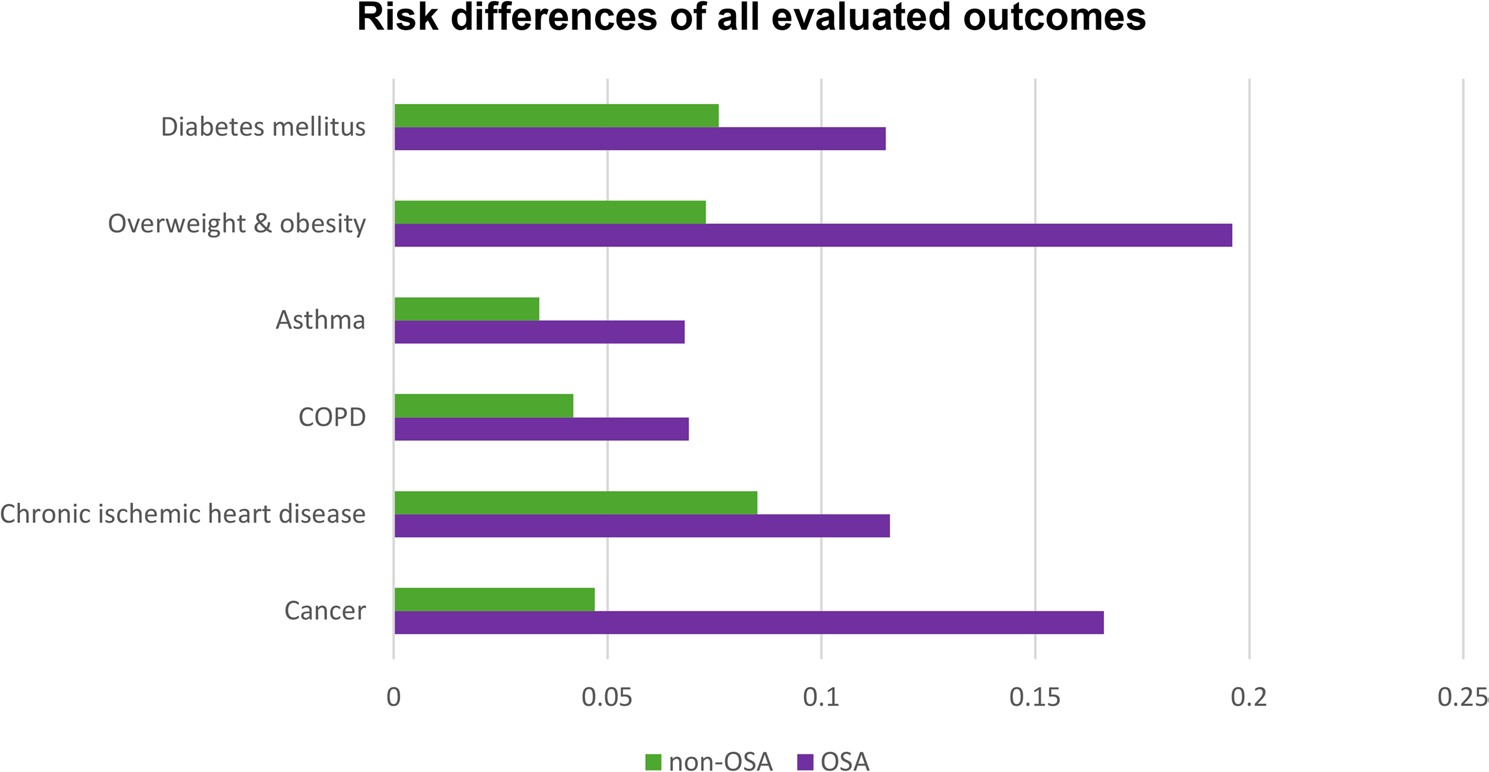
Five-Year Risk Differences for Evaluated Outcomes in Patients With and Without Obstructive Sleep Apnea. Risk differences of all evaluated outcomes for both cohorts

**Fig. 4:**
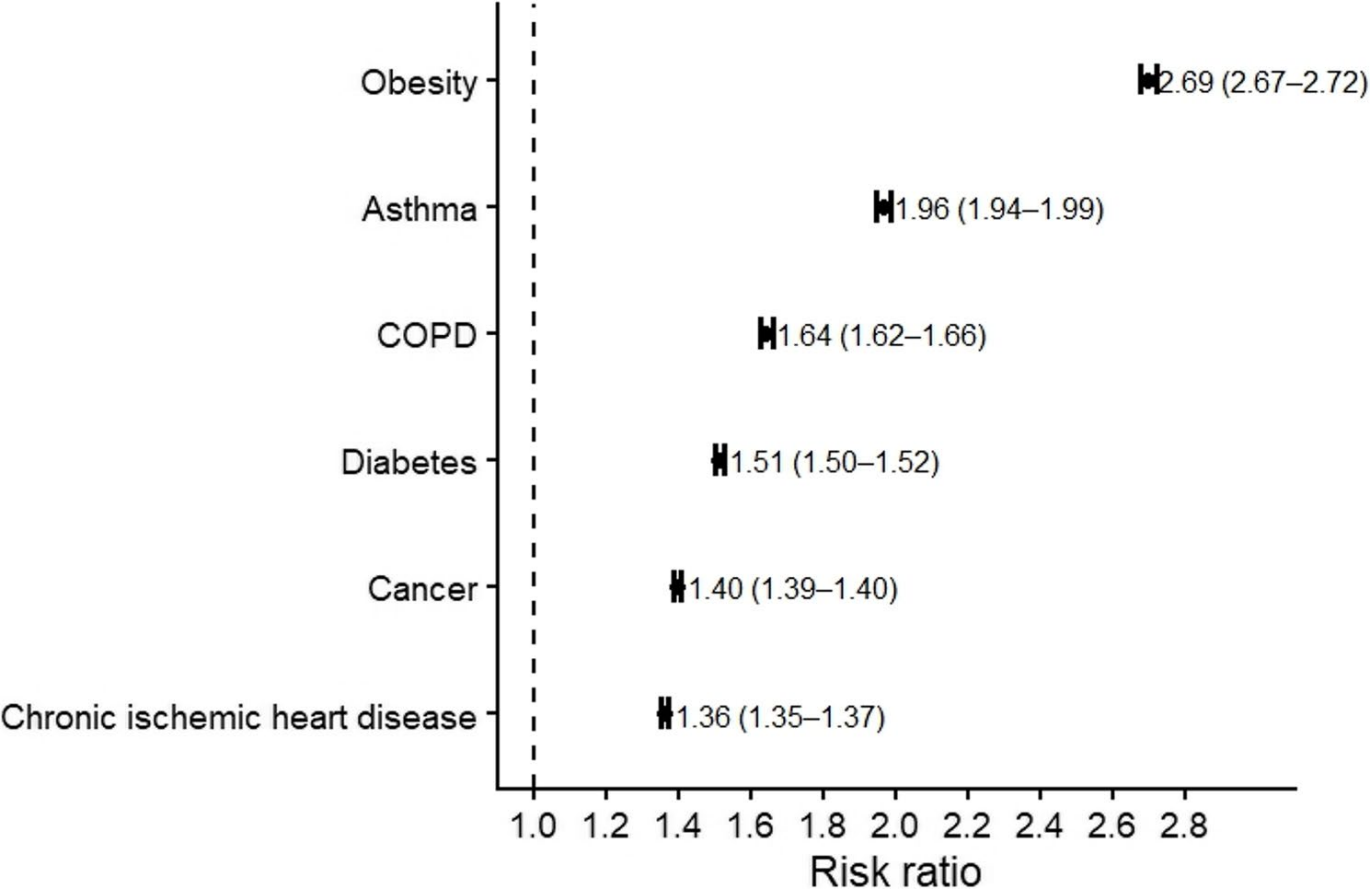
Forest Plot of Risk Ratios for Incident Outcomes in Patients With Obstructive Sleep Apnea Compared With Controls. Forest plot of univariate RRs with 95% CI for all evaluated outcomes. Points represent RRs, horizontal bars indicate 95% confidence intervals, and numeric labels denote RR (95% CI). The dashed vertical line indicates the null effect (RR = 1.0)

For chronic ischemic heart disease, 1,084,168 patients with OSA and 1,348,187 control patients were analyzed. Because of a documented diagnosis prior to the time window, 440,022 patients with OSA and 176,003 patients in the control cohort were excluded. The outcome occurred in 125,513 patients with OSA (risk 0.116) compared with 114,770 control patients (risk 0.085), corresponding to a RD of 0.031 (95% CI: 0.030–0.031; z = 79.604; *p* < 0.001), a RR of 1.360, and an OR of 1.407 (Figs. 3 and 4).

For COPD, 1,233,678 patients with OSA and 1,428,739 controls were included. Due to the outcome prior to the time window, 290,512 patients with OSA and 95,451 patients in the control cohort were excluded. COPD developed in 85,424 patients with OSA (risk 0.069) and 60,300 control patients (risk 0.042), yielding a RD of 0.027 (95% CI: 0.026–0.028; z = 96.720; *p* < 0.001), a RR of 1.641, and an OR of 1.688. (Figures 3 and 4).

For asthma, 1,200,673 patients with OSA and 1,434,421 control patients were included. Due to a documented diagnosis prior to the observation window, 323,517 patients with OSA and 89,769 patients in the control cohort were excluded. Incident asthma occurred in 81,199 patients with OSA (risk 0.068) and 49,410 controls (risk 0.034), resulting in a RD of 0.033 (95% CI: 0.033–0.034; z = 123.596; *p* < 0.001), a RR of 1.963, and an OR of 2.033 (Figs. 3 and 4).

For overweight or obesity, 703,386 patients with OSA and 1,348,995 control patients were included. Due to the outcome prior to the time window, 820,804 patients with OSA and 175,195 patients in the control cohort were excluded. The outcome occurred in 138,153 OSA patients (risk 0.196) and 98,318 controls (risk 0.073), representing the largest relative increase across all outcomes (RD 0.124; 95% CI: 0.123–0.125; z = 263.065; *p* < 0.001; RR 2.695; OR 3.109) (Figs. 3 and 4).

For diabetes mellitus, 917,274 patients with OSA and 1,282,357 controls were included. Because of a documented diagnosis prior to the observation window, 606,916 patients with OSA and 241,833 patients in the control cohort were excluded. Incident diabetes occurred in 105,229 patients with OSA (risk 0.115) and 97,293 controls (risk 0.076), yielding a RD of 0.039 (95% CI: 0.038–0.040; z = 98.258; *p* < 0.001), a RR of 1.512, and an OR of 1.578 (Figs. 3 and 4).

## Discussion

This large, real-world, propensity score–matched analysis investigated the association between obstructive sleep apnea and incident cancer as well as cardiovascular, pulmonary, and metabolic disorders in hospitalized adults using the TriNetX Global Health Research Network. Across all outcomes, patients with OSA exhibited consistently higher 5-year risks of incident comorbidities comorbidities compared with matched controls. The strongest relative risk increases were observed for overweight/obesity and asthma, followed by COPD, diabetes mellitus, chronic ischemic heart disease, and cancer, underscoring the broad systemic impact of OSA.

### Risk for incident cancer

In this analysis, OSA was associated with a significantly increased 5-year risk of incident cancer. Both risk-based and time-to-event analyses demonstrated higher cancer incidence in patients with OSA than in matched inpatients without OSA. However, the magnitude of this association was moderate and comparable to that observed for non-malignant chronic conditions, suggesting that cancer represents one component of a broader disease burden rather than a dominant downstream consequence of OSA.

Time-to-event analysis also indicated a higher cancer incidence in the OSA cohort. However, the proportional hazards assumption test was statistically significant, suggesting that the corresponding Cox model estimate should be interpreted with caution. Accordingly, greater emphasis should be placed on the overall separation of the Kaplan–Meier curves and the risk-based estimates than on the hazard ratio as a single summary measure.

Interpretation of this association remains challenging, as prior studies have yielded heterogeneous results. The existing literature can broadly be divided into two categories: experimental and epidemiological investigations. While experimental studies have consistently demonstrated biologically plausible mechanisms, epidemiological findings remain inconclusive.

From an experimental, pathophysiological perspective, several interconnected mechanisms provide biological plausibility for a link between OSA and carcinogenesis. Intermittent hypoxemia, systemic inflammation, oxidative stress, and immune dysregulation, all hallmark features of OSA, have been implicated in tumor initiation and progression. Among these mechanisms, intermittent hypoxemia appears to play a central role. Recurrent hypoxia–reoxygenation cycles activate hypoxia-inducible signaling pathways, particularly hypoxia-inducible factor 1-alpha, resulting in transcriptional programs that promote tumor progression, angiogenesis, immune modulation, and metastatic potential [33]. Upregulation of vascular endothelial growth factor (VEGF) and enhanced angiogenic signaling have been consistently observed in experimental models exposed to intermittent hypoxia [34, 35]. In vitro and animal models further show increased tumor growth, invasiveness, and metastatic spread across multiple cancer types, including head and neck, lung, breast, and liver malignancies [36, 37]. In addition, intermittent hypoxia alters the tumor microenvironment by promoting oxidative stress, systemic inflammation, and sympathetic nervous system activation. Importantly, immune evasion mechanisms appear to be critically involved, with experimental studies demonstrating impaired anti-tumor immune surveillance, increased expression of immune checkpoint molecules such as programmed death ligand 1 (PD-L1), and greater infiltration of regulatory T cells under hypoxic conditions [33, 36, 38–45]. Sleep fragmentation, another key feature of OSA, may further exacerbate these processes by promoting systemic inflammation and immune dysfunction [46, 47]. However, an important limitation of the present study is that OSA was identified through diagnostic coding only, and no information on disease severity or hypoxemia burden was available. This is particularly relevant in the context of cancer risk, as prior studies suggest that nocturnal hypoxemia and OSA severity may be more strongly associated with oncologic outcomes than the diagnosis of OSA alone.

Despite compelling mechanistic evidence, translating these findings to population-level cancer risk remains complex. Experimental models typically isolate intermittent hypoxia as a singular exposure, whereas patients with OSA exhibit a constellation of coexisting risk factors and comorbidities. In this context, epidemiological studies provide critical insight into how these mechanisms manifest clinically.

In our cohort, the parallel elevation of cancer risk alongside cardiometabolic and pulmonary diseases suggests that OSA-related biological pathways operate within a broader framework of shared risk architecture and comorbidity clustering. Hospitalized patients with OSA are characterized by advanced age, obesity, metabolic dysfunction, and chronic pulmonary disease—factors independently associated with increased cancer risk and likely to interact synergistically with sleep-disordered breathing.

From an epidemiological perspective, disentangling independent cancer risk attributable to OSA is complicated by substantial confounding from shared risk factors such as obesity, smoking, aging, and sex [44]. Several large cohort studies have shown that associations between OSA and cancer are frequently attenuated after comprehensive adjustment [8, 38, 41]. In the present study, propensity score matching explicitly accounted for male sex, thereby minimizing sex-related confounding. Despite this adjustment, an increased cancer risk persisted, indicating that the observed association cannot be explained solely by sex differences. Meta-analyses and cohort studies have suggested that female patients with OSA may experience a relatively higher risk of certain cancers compared with male patients, potentially related to differences in hormonal milieu, fat distribution, immune responses, or patterns of hypoxemia [26, 27, 33, 37, 41, 42, 46, 48]. While sex-specific effects have been suggested in prior studies, our analysis was not designed for sex-stratified inference.

Our findings are consistent with a large polysomnography - based cohort from Western Australia, which reported no independent association between OSA severity and incident cancer after adjustment for major confounders [8]. In that study, nocturnal hypoxemia was associated with cancer prevalence but not incidence, suggesting that hypoxia-related mechanisms may reflect comorbidity clustering rather than direct oncogenesis. Although methodological approaches differ, both studies converge on the interpretation that OSA is unlikely to function as a cancer-specific driver. Meta-analyses support a modest overall increase in cancer incidence among patients with OSA, particularly in studies incorporating objective hypoxemia measures [49]; however, these associations are often weakened after adjustment. Notably, not all large registry-based studies have demonstrated increased cancer-related mortality in OSA populations [39].

Taken together, epidemiological evidence supports a modest association between OSA and cancer risk that appears to be strongly shaped by shared risk factors and comorbidity patterns. In line with our findings, cancer should be viewed as one element within a broader spectrum of long-term morbidity associated with OSA rather than as a distinct or disproportionate outcome.

### Riks for cardiovascular, pulmonary, and metabolic disorders

Beyond cancer, OSA was associated with substantially increased 5-year risks of cardiovascular, pulmonary, and metabolic disorders. Elevated risks of chronic ischemic heart disease and diabetes mellitus are consistent with extensive prior literature linking OSA to cardiometabolic dysfunction through sympathetic activation, endothelial dysfunction, intermittent hypoxemia, systemic inflammation, and metabolic dysregulation [6, 16–18, 45, 50, 51]. Importantly, these associations were comparable to or stronger than those observed for cancer, highlighting that non-malignant diseases constitute the predominant long-term burden of OSA.

Metabolic outcomes demonstrated the strongest relative associations, particularly for overweight and obesity. This finding should be interpreted with caution, as obesity is a major risk factor for OSA and was not accounted for in the matching process. Therefore, the observed effect estimate may partly reflect residual confounding and shared risk architecture rather than a direct effect of OSA on incident overweight or obesity. While prior literature suggests a potentially bidirectional relationship between OSA and metabolic dysfunction, the present analysis does not permit causal interpretation [45, 52, 53].

Pulmonary outcomes showed particularly strong associations. Patients with OSA had markedly higher risks of incident COPD and asthma, extending existing knowledge beyond cross-sectional descriptions of overlap syndrome and asthma–OSA coexistence [54–58]. While shared risk factors such as smoking and obesity are likely to contribute, the persistence of these associations after matching suggests that sleep-disordered breathing may exacerbate or unmask underlying pulmonary vulnerability. Recurrent nocturnal hypoxemia, airway inflammation, and altered respiratory mechanics during sleep may accelerate chronic airway disease progression [58–60]. Notably, robust 5-year incidence estimates for asthma and COPD in OSA populations have been largely absent from prior literature, which has predominantly focused on prevalence or reverse associations [54].

Collectively, these results reinforce the concept of OSA as a systemic disorder with wide-ranging and sustained health consequences. The parallel elevation of cardiometabolic, pulmonary, and oncologic risks indicates that OSA identifies a high-risk phenotype characterized by multisystem morbidity rather than a condition associated with a single dominant downstream outcome. Clinically, these findings emphasize the importance of comprehensive risk stratification and longitudinal management strategies for patients with OSA, particularly in hospitalized populations with substantial baseline disease burden. Another important consideration is potential detection bias. Patients with OSA may have more frequent healthcare contacts, diagnostic evaluations, and follow-up care than patients without documented OSA. This increased surveillance may have facilitated earlier or more frequent detection of incident conditions, including cancer and chronic cardiometabolic or pulmonary diseases. Therefore, part of the observed excess risk may reflect differences in healthcare utilization and disease ascertainment rather than true differences in underlying incidence.

### Limitations

Several limitations should be acknowledged. First, misclassification and detection bias cannot be fully excluded. Diagnostic accuracy and coding practices may vary across institutions, and patients with OSA may undergo more frequent medical evaluations and diagnostic testing, increasing the likelihood that incident diseases are identified during follow-up. As a result, some of the observed associations may partly reflect differences in healthcare utilization and disease ascertainment rather than true differences in disease occurrence. In addition, key sleep-specific variables and important lifestyle-related confounders, including smoking intensity, physical activity, and diet, are not consistently captured in structured electronic health record data. Propensity score matching was restricted to age and sex, while other relevant clinical and lifestyle-related confounders, such as obesity, smoking, alcohol use, baseline metabolic status, and comorbidity burden, were not included in the matching process. Accordingly, residual confounding cannot be excluded and may have influenced the reported effect estimates. This is particularly relevant for the overweight/obesity outcome, as obesity is closely linked to both the development and diagnosis of OSA.

Second, outcomes were assessed over a 5-year follow-up period, which may be insufficient to capture long-latency processes such as carcinogenesis for certain tumor types. In addition, OSA was identified using ICD-10 coding only, which may have introduced diagnostic misclassification, and no information on disease severity, hypoxemia burden, or treatment status was available. Therefore, severity-dependent associations and potential dose–response relationships could not be examined. Moreover, OSA status and treatment exposure were modeled as baseline variables, although both may change over time, potentially introducing unmeasured time-varying confounding.

Finally, undiagnosed OSA in the control group represents a further source of bias. Because OSA is frequently underdiagnosed, some individuals classified as controls may have had unrecognized disease. In addition, both cohorts were restricted to hospitalized patients, and the study population may therefore not be representative of the broader OSA population. This may have introduced selection bias and limits the generalizability of the findings, particularly to patients with milder disease managed exclusively in outpatient settings.

These limitations should be considered when interpreting the magnitude and generalizability of the observed associations.

## Conclusions

In this large, real-world, propensity score–matched analysis of hospitalized patients, OSA was associated with increased 5-year risks of incident cancer as well as cardiovascular, pulmonary, and metabolic diseases. Importantly, the magnitude of cancer risk was comparable to that observed for several non-malignant chronic conditions, reinforcing the concept of OSA as a systemic disorder characterized by sustained multisystem morbidity rather than a cancer-specific disease entity. These findings suggest that the relationship between OSA and cancer is embedded within a broader framework of shared risk factors and comorbidity clustering, rather than reflecting a uniform or independent oncogenic effect of sleep-disordered breathing. From a clinical and public health perspective, our results underscore the importance of comprehensive risk stratification and long-term management strategies for patients with OSA that extend beyond cancer surveillance to include cardiometabolic and pulmonary disease prevention. These findings should be interpreted in the context of the hospitalized study population and may not be fully generalizable to broader, non-hospitalized populations. Future prospective studies incorporating detailed sleep metrics, longitudinal treatment data, and refined confounder control are warranted to further clarify the independent contribution of OSA to long-term health outcomes.

## Data Availability

The data that support the findings of this study are available from the corresponding author upon reasonable request.

## Abbreviations

OSA: Obstructive sleep apnea
COPD: Chronic obstructive pulmonary disease
RR: Risk ratio
BMI: Body mass index
HCO: Health care organization
HIPAA: Health Insurance Portability and Accountability Act
95% CI: 95% confidence intervals
RD: Risk difference
OR: Odds ratio
VEGF: Vascular endothelial growth factor
PD-L1: Programmed death ligand 1
